# Serum Insulin-Like Growth Factor-1 in Patients with De Novo, Drug Naïve Parkinson’s Disease: A Meta-Analysis

**DOI:** 10.1371/journal.pone.0144755

**Published:** 2015-12-11

**Authors:** Dun-Hui Li, Ya-Chao He, Thomas J. Quinn, Jun Liu

**Affiliations:** 1 Department of Neurology & Institute of Neurology, Ruijin Hospital Affiliated to Shanghai Jiao Tong University School of Medicine, Shanghai, 200025, China; 2 Department of Radiation Oncology, William Beaumont Hospital, Royal Oak, Michigan, 48073, United States of America; Philadelphia VA Medical Center, UNITED STATES

## Abstract

**Objective:**

Insulin-like growth factor-1 (IGF-1) is reported to be neuroprotective in the setting of Parkinson’s disease (PD), and there is increasing interest in the possible association of serum IGF-1 levels with PD patients, but with conflicting results. Therefore, we conducted a meta-analysis to evaluate the association of serum IGF-1 levels in de novo, drug naïve PD patients compared with healthy controls.

**Methods:**

Pubmed, ISI Web of Science, OVID, EMBASE, and Cochrane library databases from 1966 to October 2014 were utilized to identify candidate studies using Medical Subjective Headings without language restriction. A random-effects model was chosen, with subgroup analysis and sensitivity analysis conducted to reveal underlying heterogeneity among the included studies.

**Results:**

In this meta-analysis, we found that PD patients had higher serum IGF-1 levels compared with healthy controls (summary mean difference [MD] = 17.75, 95%CI = 6.01, 29.48). Subgroup analysis demonstrated that the source of heterogeneity was population differences within the total group. Sensitivity analysis showed that the combined MD was consistent at any time omitting any one study.

**Conclusions:**

The results of this meta-analysis demonstrate that serum IGF-1 levels were significantly higher in de novo, drug-naïve PD patients compared with healthy controls. Nevertheless, additional endeavors are required to further explore the association between serum IGF-1 levels and diagnosis, prognosis and early therapy for PD.

## Introduction

Parkinson’s disease (PD) is the second most common neurodegenerative disease, and is characterized by bradykinesia, resting tremor, rigidity and postural instability. The morbidity of this chronic progressive disorder is anticipated to rise as the affected population continues live longer and increase in number.[1] Although the etiology of PD remains obscure, oxidative stress appears to play an important role in the progression of PD,[2] which results in severe degeneration and loss of dopaminergic neurons in the substantia nigra pars compacta, with subsequent development of PD.[3] Insulin-like growth factor-1 (IGF-1) is a 70-amino acid polypeptide chain that plays a critical role in regulating cellular function, metabolism, survival and differentiation.[4] The protective effect of IGF-1 against dopamine induced neurotoxicity was demonstrated in human and rodent cell cultures.[5] Moreover, in cell models of PD, IGF-1 was found to protect SH-EP1 cells from 1-methyl-4-phenylpyridinium (MPP^+^) induced apoptotic cell death[6] and augmented cellular antioxidant defense mechanisms through up-regulation of heme oxygenase-1 (HO-1) expression,[7] which may provide effective protection against dopaminergic neuron loss. Furthermore, behavioral recovery was observed after peripheral administration of IGF-1 in a 6-hydroxydopamine (6-OHDA) lesioned rat model of PD.[8] Indeed, a number of recent investigations have been conducted to evaluate serum IGF-1 levels among de novo, drug-naïve Parkinson’s disease patients versus healthy controls. Nevertheless, the results from these studies are not entirely consistent.[9–13] Therefore, a comprehensive evaluation of serum IGF-1 levels in PD patients is necessary. To that end, the purpose of this study was to evaluate the existing literature regarding serum IGF-1 levels in de novo, drug-naïve PD patients in comparison with healthy controls, and synthesize a thorough meta-analysis which may facilitate future investigations into novel ways to diagnose, estimate prognosis and initiate early therapy in patients with PD.

## Materials and Methods

### Literature search

Our study was performed according to the Preferred Reporting Items for Systematic Reviews and Meta-analyses (PRISMA)[14] (S1 Checklist). We searched five major electronic databases: Pubmed, ISI Web of Science, OVID, EMBASE, Cochrane library databases and reference lists up to October 2014 without language restriction. All inquiries utilized Medical Subject Headings (MeSH) with the following keywords: “insulin-like growth factor-1” or “Parkinson’s disease”. All articles and correlative references were evaluated for relevance to serum IGF-1 and de novo, drug-naïve PD patients. We also tried to acquire unpublished and negative results through searching the International Standard Randomized Controlled Trial Number (ISRCTN) registry and the International Clinical Trials Registry Platform (ICTRP) search portal, but no relevant studies were identified. Two authors independently performed the above literature search, with any questionable studies discussed and evaluated in detail.

### Inclusion criteria

The eligibility of articles included in this meta-analysis were evaluated by the following inclusion criteria: (1) case-control studies comparing serum IGF-1 levels between de novo, drug-naïve idiopathic PD patients and healthy controls, or cohort studies with detailed baseline data; (2) the diagnosis of PD must be made according to the UK Parkinson’s Disease Society Brain Bank[15]; (3) detailed methods for detecting serum IGF-1 must be available; (4) definite serum IGF-1 mean and SD values must be reported. Furthermore, two authors independently evaluated the eligibility of all identified papers based on the above inclusion criteria. Ultimately, five studies were identified and included in our meta-analysis.

### Exclusion criteria

Review articles, commentaries, and conference proceedings without new data were excluded from this meta-analysis. Additionally, all articles pertaining to the fundamental pathophysiology of IGF-1 as related to the pathogenesis or treatment of PD were excluded. Other studies were excluded if their participants were diagnosed with secondary parkinsonism, atypical parkinsonism, familial parkinsonism or suffered from any disease that may affect serum IGF-1 levels. Two authors independently screened all articles based on the above exclusion criteria.

### Quality assessment, data extraction and analysis

The Newcastle-Ottawa Scale (NOS) criteria,[16] including the selection (0–4 points), comparability (0–2 points) and exposure (0–3 points) categories, were applied to assess the quality of all included studies, with a higher score indicating higher quality methodology. The following data were extracted from the five included studies: name of first author, year of publication, number of de novo drug-naïve PD patients and healthy controls, and the mean and standard deviation (SD) of serum IGF-1 levels. Data analysis was conducted with Cochrane Collaboration software (Review Manager 5.3) and 95% confidence intervals (CI) and mean difference (MD) were calculated. Heterogeneity, subgroup and sensitivity analyses were also carried out with Review Manager 5.3 software.

## Results

### Literature search and study characteristics

**Fig 1:**
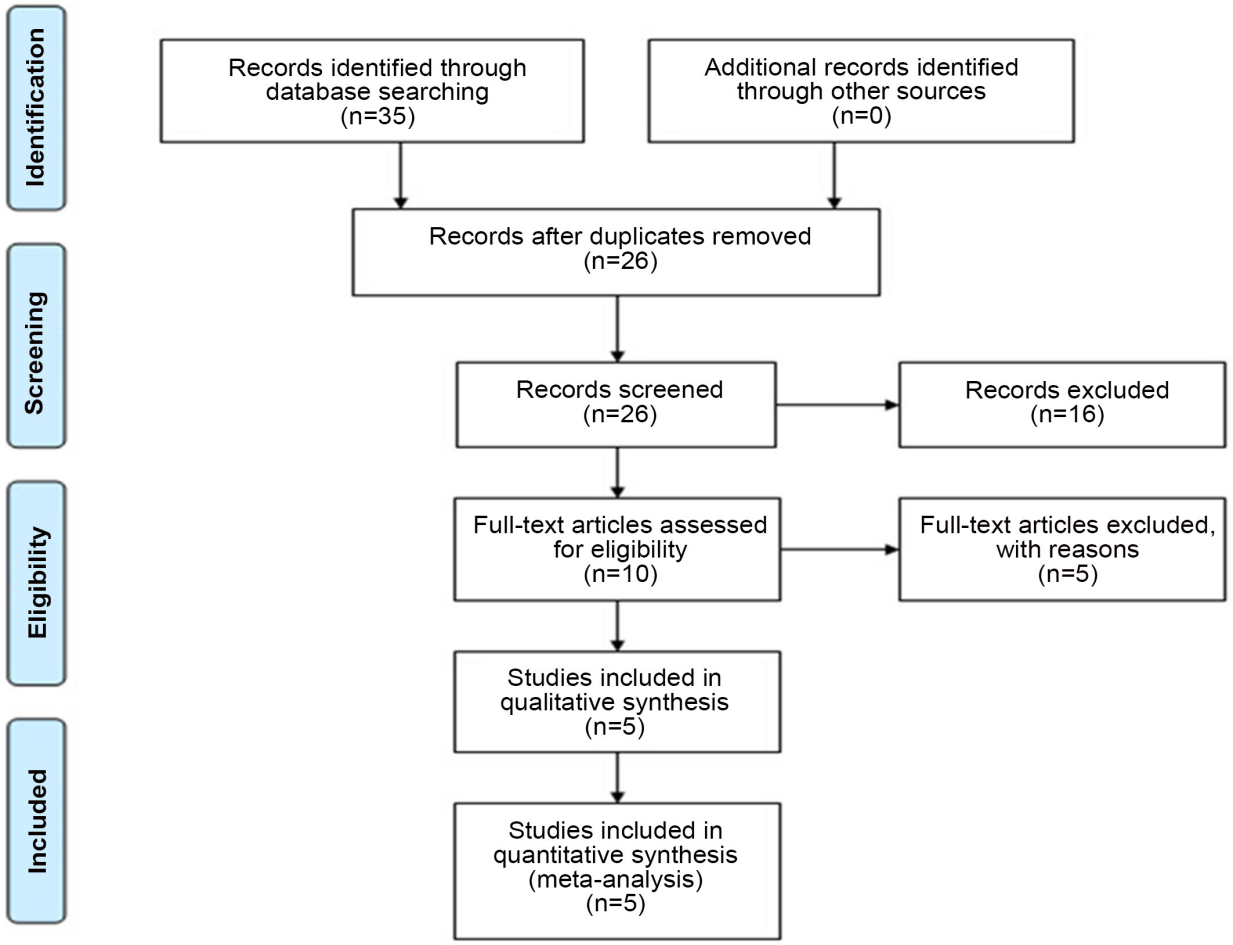
Selection process of included studies in the present meta-analysis

Initially, 35 studies were retrieved with MeSH words: “Parkinson’s disease” and “insulin-like growth factor-1”. After reviewing the titles and abstracts, 9 replicate studies, 8 fundamental research papers, 2 reviews and 6 other articles irrelevant to serum IGF-1 levels in PD patients were eliminated. Of the remaining 10 papers, 2 commentary papers, 1 article with no de novo drug-naïve PD patients, 1 article with plasma IGF-1 and 1 study investigating the IGF-1 gene were excluded. Ultimately, 5 studies with 166 PD patients and 323 healthy controls were included in our meta-analysis. Of these 5 studies, 2 were conducted in Germany, 2 were carried out in Italy and 1 was conducted in Japan. The selection process is further detailed in Fig 1. The characteristics of the 5 studies are detailed in Table 1. The overall quality of the included studies was good as assessed by Newcastle-Ottawa Scale (NOS) criteria (Table 2).

**Table 1:** Characteristics of included studies.

Author Name	Year	Country	N		Mean±SD of serum IGF-1			PD Definition	Variables controlled
			PD	CON.	PD	CON.	P value		
Godau	2010	Germany	6	12	149±30.3	98.9±23.2	<0.001	UK Brain Criteria	BMI>30, diabetes, cancer, thyroid disorders, dementia, chronic disease, any medication
Godau	2011	Germany	15	139	158.4±40.4	126.6±31	0.004	UK Brain Criteria	BMI>30, diabetes, pitutiary of thyroid disease, chronic inflammatory disease, Cancer, use of β-blocker, corticosteroid, neuroleptic of hormone replacement medication
Picillo	2013	Italy	55	60	94.5±37.5	79.1±23	<0.011	UK Brain Criteria	Secondary, familial or atypical parkinsonism, diabetes, obesity, pituitary or thyroid disease, acute or chronic inflammatory disease, cancer
Pellecchia	2012	Italy	65	60	91.6±34.4	79.1±23	0.019	UK Brain Criteria	atypical, secondary, familial or iatrogenic parkinsonism, dementia, major depressive disorder, disorder interfering serum IGF-1
Numao	2014	Japan	25	52	118.1±8.4	114.4±5.9	>0.05	UK Brain Criteria	BMI>30, diabetes, acromegaly, neurological disorders, dementia, psychiatric disorders, thyroid diseases

N: Number;

PD: Parkinson’s disease;

CON.: Healthy Controls.

**Table 2:** Quality assessment of included studies based on the Newcastle-Ottawa Scale.

Author and Year	Selection	Comparability	Exposure
Godau 2010	3	2	3
Godau 2011	3	1	3
Picillo 2013	4	2	2
Pellecchia 2014	4	2	2
Numao 2014	3	2	3

The Newcastle-Ottawa Scale (NOS) criteria is composed of 3 major categories: selection (4 items), comparability (1 item) and exposure (3 items). Each item in the selection and exposure section can be awarded a maximum of one point, with a maximum of two points in the comparability section. A higher score indicates higher quality methodology.

### Serum insulin-like growth factor-1 in Parkinson’s disease patients

**Fig 2:**
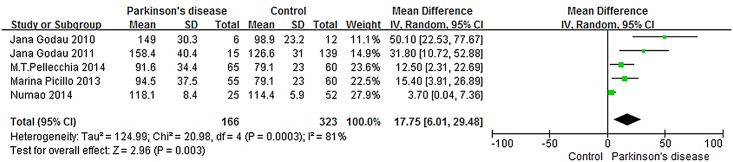
Serum IGF-1 levels in PD patients compared with healthy controls.

The random-effects model was used to calculate the pooled effect size of the five studies. All available data from the five included studies were used to compare different serum IGF-1 levels between de novo, drug naïve PD patients and healthy controls. Notably, a significant difference was found between the two groups, and the pooled mean difference in the random-effects model was 17.75, 95%CI: 6.01, 29.48 (Fig 2).

### Sensitivity analysis and subgroup analysis

**Fig 3:**
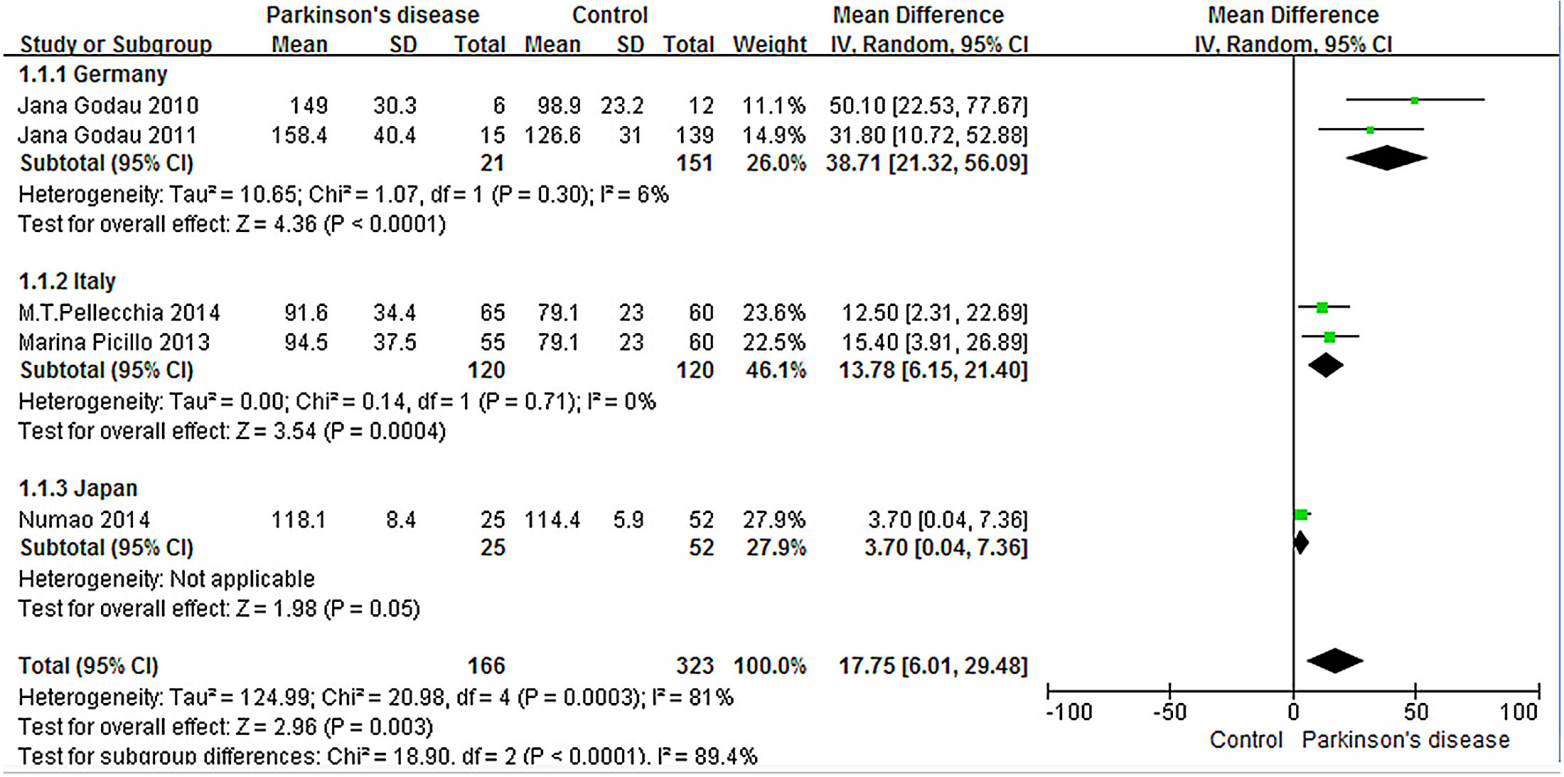
Population-based subgroup analysis of serum IGF-1 levels in PD patients compared with healthy controls.

Sensitivity analysis was performed to evaluate the stability and reliability of our meta-analysis by repeating analysis in various combinations of the included studies in which a single study is omitted during each reanalysis. No substantial alteration of the results were found in this sensitivity analysis, with the combined mean difference (MD) ranging from 12.70 (95% CI, 2.99, 22.41) via omission of the research by Godau 2010, to 22.72 (95% CI, 9.81, 35.64) via omission of the research by Numao 2014, indicating statistically robust results. Next, subgroup analysis was carried out to assess for heterogeneity within this meta-analysis. Due to variations among different populations’ serum IGF-1 levels, this meta-analysis is highly heterogeneous (I^2^ = 81%, P<0.0001). Therefore, we divided the total group into three subgroups according to the nation where the research was performed: Germany (N = 2), Italy (N = 2), and Japan (N = 1). These three subgroups resolved the overall heterogeneity, with study heterogeneity no longer significant in the Germany subgroup (I^2^ = 6%, P = 0.30) or the Italy subgroup (I^2^ = 0%, P = 0.71). Heterogeneity analysis is not applicable to the single study in the Japan subgroup. The various subgroups are represented by forest plots in Fig 3.

## Discussion

In this meta-analysis comparing different serum IGF-1 levels between de novo, drug naïve PD patients and healthy controls, we found significantly higher serum IGF-1 levels among PD patients. Although initial analysis revealed a significant degree of heterogeneity among the included studies, subgroup analysis reasonably addressed this problem by categorizing the five studies by country of origin. Unfortunately, there are no reports discussing variations in serum IGF-1 levels among German, Italian and Japanese populations. Nevertheless, population differences have been demonstrated, with significantly higher mean IGF-1 levels in healthy African-Americans compared with the Japanese population.[17] Indeed, these population variations may account for the obvious heterogeneity in our meta-analysis when calculating the pooled effect size. Another source of heterogeneity may be due to variations in detecting serum IGF-1. However, four different methods, including radioimmunoassay, immunoradiometric assay, and immunoassay systems, were used in the five primary studies, which makes it unlikely to contribute to the observed heterogeneity.

Among the five studies included in our investigation, three studies explored the relationship between serum IGF-1 levels and motor impairment, but with controversial results. Godau et al[11] found that patients with the highest serum IGF-1 levels had low UPDRS-III scores, a strong inverse correlation. However, in a 2-year follow-up study based on the Italian population, patients with the highest serum IGF-1 quartile at baseline showed significantly higher UPDRS-III scores[12]. Moreover, the study found that dopaminergic score rose with the IGF-1 quartile as well, which was rationalized by proposing that high IGF-1 levels at diagnosis might indicate patients with severe motor symptoms and, therefore, these patients might respond well to dopaminergic administration in the early stage of the disease.[12] However, no significant correlation between serum IGF-1 levels and UPDRS-III score was found in the remaining Japanese study.[13] The variation in outcome in each study may be attributed to inherent differences among the populations studied or might be due to inadequate sample size within each study. Therefore, to confirm whether high serum IGF-1 levels at early stages of PD is a compensatory response to neuronal degeneration or a susceptibility marker of the degenerative progress, larger cohort studies are needed.

In contrast to the controversial correlation between serum IGF-1 levels and motor dysfunction in PD patients, a recent study concluded that among the elderly population, higher serum IGF-1 levels were associated with greater cognitive function, including: working memory, selective attention and executive function.[18] In addition, a deficiency of IGF-1 among the elderly (<9.4nmol/l) may influence the level and rate of decline of information process speed.[19] In a study by Pellecchia et al[10], low serum IGF-1 in de novo, drug-naïve PD patients at baseline was associated with poor performance on cognitive function after a 2 year follow-up. Therefore, we propose that a low IGF-1 level at baseline might serve as a serum biomarker for cognitive decline in PD patients through further cohort studies.

To our knowledge, this is the first meta-analysis comparing serum IGF-1 levels between PD patients and healthy controls. Our results demonstrate that serum IGF-1 levels are significantly higher in a de novo, drug-naïve PD patient cohort, which bolsters the hypothesis that elevated serum IGF-1 levels may act as a biomarker for the early diagnosis of PD; however, well-designed clinical investigations evaluating sensitivity, specificity and predictive value in the diagnosis of PD must be done to further test this hypothesis. Furthermore, additional research is necessary to verify whether high serum levels of IGF-1 at baseline could predict better outcomes during disease progression[11] or indicate severe motor dysfunction[12]. In addition, the correlation of IGF-1 levels with other clinical presentations, e.g., cognitive decline, hyposmia, psychological symptoms, or rapid eye movement sleep behavior disorder (RBD), remain to be explored. Indeed, a recent animal study found that depressed rats had significantly reduced serum IGF-1 levels.[11]

There are several key limitations to this meta-analysis. First, this meta-analysis was based on observational inspection, therefore, potential bias or confounding in these studies must be considered. Second, we were unable acquire unpublished data or studies with negative results, resulting in a degree of publication bias within the included studies. Thirdly, although a thorough research algorithm was used to acquire the published data, publication bias may confound the current study. Analysis of funnel plots proposed by Egger[20] could be a useful test to assess potential publication bias; however, according to the Cochrane Handbook, application of this method with fewer than 10 studies would be unwise.[21] Therefore, funnel plot analysis was not utilized in this study. In addition, since the original studies lacked gender-specific data, we were unable to conduct further analysis stratified by sex. Previous research found that estrogen can interact with IGF-1, providing neuroprotection to nigrostriatal neurons[22]. Moreover, estrogen can enhance transcription by interacting with enhancer response elements (EREs) and growth hormone-responsive element (GHREs) of the IGF-1 gene.[23] In addition, age has been reported to be inversely correlated with serum IGF-1[10], however, we did not acquire any age-specific data from the included studies, further confining our meta-analysis.

## Conclusions

Our meta-analysis provides evidence that de novo, drug-naïve PD patients have significantly higher serum IGF-1 levels when compared with healthy controls. Further studies should be carried out to verify these results among different populations and a well-designed cohort study should be carried out to evaluate the correlation between baseline serum IGF-1 and motor function among PD patients. In addition, the pathophysiology and biochemical mechanism of higher serum IGF-1 in PD patients could be further investigated with the hopes of unveiling potential therapeutic targets within the IGF-1 pathway.

## Supporting Information

S1 Checklist PRISMA 2009 checklist used in this meta-analysis. (DOC)

## Abbreviations

CI: Confidence interval
EREs: Enhancer response elements
GHREs: Growth hormone-responsive elements
HO-1: Heme oxygenase-1
IGF-1: Insulin-like growth factor-1
MD: Mean difference
MeSH: Medical subject headings
MPP^+^: 1-methyl-4-phenylpyridinium
NOS: Newcastle Ottawa Scale
PD: Parkinson’s disease
RBD: Rapid eye movement sleep behavior disorder
SD: Standard deviation
UPDRS: Unified Parkinson’s disease Rating Scale
